# Proximity to Practice: The Role of Technology in the Next Era of Assessment

**DOI:** 10.5334/pme.1272

**Published:** 2024-12-26

**Authors:** Andrew E. Krumm, Hollis Lai, Kayla Marcotte, Tavinder K. Ark, Victoria Yaneva, Saad Chahine

**Affiliations:** 1 Learning Health Sciences, Surgery, and Information, Medical School and School of Information, University of Michigan, Ann Arbor, Michigan, United States; 2 Faculty of Medicine and Dentistry, University of Alberta, Canada; 3 Department of Learning Health Sciences, University of Michigan, Ann Arbor, Michigan, United States; 4 Data Science Institute and Center for Advancing Population Science, Medical Colleges of Wisconsin, United States; 5 NBME, Philadelphia, United States; 6 Faculty of Education, Queen’s University, Canada

## Abstract

The integration of technology into health professions assessment has created multiple possibilities. In this paper, we focus on the challenges and opportunities of integrating technologies that are used during clinical activities or that are completed by raters after a clinical encounter. In focusing on technologies that are more proximal to practice, we identify tradeoffs with different data collection approaches. To maximize the benefits of integrating technology in workplace-based assessment, we describe the importance of using preexisting frameworks from the fields of assessment design, implementation research, and clinical artificial intelligence governance.

Assessment and technology are intertwined. Simulations, intelligent tutors, computer adaptive tests, and automated item generation are among the many examples of how assessment and technology are connected in health professions education [HPE; 1,2,3]. Recently, generative artificial intelligence and large language models have led to new ways of collating and summarizing assessment data [4]. While there are multiple ways in which technology influences assessment systems, one increasingly important way entails using technology to collect assessment evidence more directly from clinical practice. Collecting data that is proximal to where and how actual clinical activities are carried out creates opportunities but also poses several challenges. For example, the factors that make the clinic, ward, or operating room a critical assessment setting can also make it harder to isolate what a learner knows and can do from the myriad factors influencing a learner’s performance [5,6]. Therefore, a key goal for the next era of assessment involves collecting data from practice in ways that can support sound assessment claims about a learner.

There is a robust history of using data from practice for assessment purposes [7,8]. When using data directly from practice, important questions resurface that are often taken for granted with traditional test-based assessments. To address these questions in principled ways, multiple frameworks exist to support both development and use of an assessment system [9]. In this Eye Opener, we highlight three frameworks that can be used to get the most out of data gathered directly from clinical activities: Evidence Centered Design (ECD), implementation research, and artificial intelligence governance [9,10]. Our goal in this paper is to introduce these frameworks and how they can enable better uses of technology in collecting data from clinical environments for assessment purposes.

## The Challenges of Authenticity

Assessment evidence collected directly from the workplace often bring with them the label of being more “authentic” than data collected through assessment modalities like multiple-choice tests. Because workplace tasks take place over time, both the activities one engages in and/or the products of those activities can be the target of assessment. In the case of procedure-based medical specialties, for example, both the ways in which the procedure is carried out and the outcome of a procedure can be evaluated [11,12]. When the purpose of gathering assessment evidence is to measure latent abilities of a learner, issues of replicability and generalizability of the tasks from which data are collected come to the fore [13,14,15]. As Messick argues, “…consistency or variability of the performances contributes to score meaning, as does generalizability from the sample of observed tasks to the universe of tasks relevant to the knowledge of skill domain at issue” [8]. Thus, as the object of measurement centers on learners’ more general knowledge, skills, and attributes, integrating existing reliability and validity standards with new and novel sources of evidence becomes a central issue to address.

Messick further argues that despite the seeming benefits of collecting more authentic assessment evidence, the implication of authenticity does not remove the need to address two components of construct invalidity: irrelevant variance and underrepresentation [8]. Sources of irrelevant variance like varying complexity of patients’ health-related issues as well as differing routines abound when assessing healthcare professionals in the workplace [16]. Construct underrepresentation can occur when a summary score from an assessment process does not include evidence from clinical tasks that are considered central to the construct [8]. For example, a surgeon’s overall operative performance could be underrepresented if only performances for one procedure are used to calculate an overall operative performance score.

While the increasing use of technology in healthcare settings has made the collection of data from practice easier, the degree to which data are considered better or higher quality depends upon more than the location from which they were collected. The object of measurement (i.e., point-in-time task performance or latent construct) as well as the stakes of the decision to be supported by a resulting score all help in characterizing the quality of the evidence. In many ways, new data brings back old considerations of what makes *any* data good for assessment. While there is utility in returning to fundamentals around new advances, researchers have regularly developed strategies for closing the gap between what is considered “old” and “new” at a given point in time. For example, researchers have developed approaches for using data generated from simulations and game-based environments that have advanced not only how data are analyzed but how to strengthen assessment arguments around data collected from rich learning environments more generally [17]. As researchers have worked to understand how assessment data can be used to drive decision-making, barriers and facilitators to high quality implementation have emerged [18]. Lastly, as more and more tools are developed to algorithmically guide or enact decisions based on digital data sources, the ways in which algorithms are governed and evaluated have continued to evolve [19]. To help researchers work with data gathered from practice using various technologies, we next highlight three frameworks that could guide future research and development activities.

## Frameworks

Key resources for assessment users, assessment developers, and HPE leaders are frameworks that can facilitate the potential adoption and implementation of technology-based assessments. At a surface level, frameworks can serve as checklists, helping individuals remember to ask or investigate key topics. More deeply, frameworks can also help in providing a comprehensive mental model for making sense of seemingly discrete, disconnected decisions. In the case of assessment development, certain frameworks can help in spanning the two quite distinct phases that accompany any measurement process—development and use [20].

Measurement development involves creating an assessment argument about how the data gathered from a focal task is indicative of high or low performance for a desired target of measurement. In addition, development entails building data collection instruments and generating the kinds of reliability and validity evidence that would help a future user of an assessment decide to deploy it. Use, by contrast, activates a different and more operational set of activities: How will data be collected in line with ongoing work and patient care activities? How will data be summarized in near real-time? and How will resulting summary statistics be communicated to the right person at the right time in the right format in support of specific educational decisions and interventions? Traversing the complexities of both development and use requires more than a single framework. Moreover, when the settings in which data to be collected for assessment purposes are not under the direct control of end users like a standardized multiple-choice test by having learners literally remove themselves from professional activities, a broader set of implementation factors and consequences need to be considered. Below, we highlight three frameworks organized around assessment development, implementation research, and governance that can aid in navigating the intersection of technology and assessment when working with data that is drawn more directly from clinical activities.

**Figure 1:**
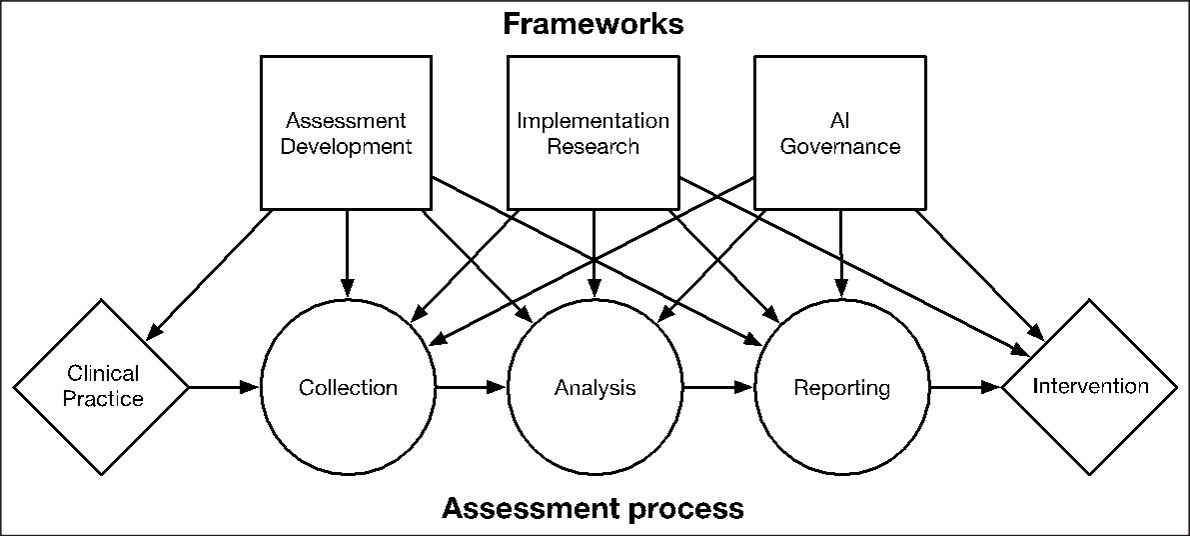
Alignment between frameworks and generic assessment process. **Note:** Arrows between Frameworks (top) and Assessment process (bottom) demonstrate the most important links between a framework and a process.

Figure 1 provides an overview of the ways in which assessment development, implementation research, and governance affect the generic assessment processes of collecting, analyzing, and reporting on data. The assessment process outlined in Figure 1 is organized around data that originates from Clinical Practice and ends with an educational Intervention (e.g., remediate, accelerate, or certify a learner). Frameworks associated with Assessment Development can help in identifying important clinical practices to assess, while building validity arguments that span data collection to reporting. Implementation Research can help in identifying the barriers and facilitators of putting into place assessment systems and the changes in organizational behavior needed to get the most out of the system. Lastly, AI Governance addresses the educational interventions and their evaluation.

### Assessment Development

Assessment development can involve various “layers” [21]. From the perspective of Evidence-Centered Design (ECD), development entails (1) understanding the object of measurement, (2) developing a coherent argument that connects the object of measurement to tasks that elicit that object of measurement based on evidence generated from a focal task; (3) developing operational algorithms, (4) gathering evidence through implemented tasks, and (5) scoring and reporting on collected evidence. The layers of ECD offer a coherent framework for developing assessment arguments and connecting those arguments to data collection, analysis, and reporting activities [22].

ECD helps to address construct irrelevant variance and underrepresentation both descriptively and operationally. Descriptively, ECD can help in documenting the choices that assessment developers make. Within the second layer mentioned above, which is often referred to as “domain modeling,” assessment developers define a “student model” as the targeted proficiencies [21]. Quality definitions include not just what is intended to be measured but the additional knowledge and skills that could be measured from a task and contribute irrelevant variance [21]. As part of domain modeling, the “task model” defines features of the clinical activity from which data will be collected; the task model also helps in isolating what will be attended to from the clinical activity and why. That which is attended to is then used to generate a score, where high or low performance is defined statistically in the “evidence model.” If student, task, and evidence models cannot be defined and the connections across the three models are not coherent, then the initial, more descriptive layers of ECD have done their job in highlighting potential issues with data drawn directly from a clinical activity.

ECD, however, does not just address descriptive elements of an assessment argument; it also helps in defining operational elements. These operational elements include (1) selecting an activity to assess, (2) providing a way of collecting evidence from that activity (e.g., a rating form), (3) evaluating evidence collected from a focal activity, and (4) summarizing multiple sources of evidence if an object of measurement requires multiple observations [9]. In graduate medical education, many assessments of what a learner can do are rater-mediated. Smartphones make it easy to collect structured observational data of a learner performing a clinical task [23]. For smartphone-based, rater-mediated systems, operational issues involve addressing the ways in which a rater selects from a list of clinical activities on which to assess a learner. Additional issues include presenting a rater with observation prompts and one or more rating scales; most systems end the assessment process after evidence has been collected and processed for a single assessment encounter. The additional operational step of summarizing multiple ratings highlights an often missed opportunity of being able to better represent a construct by including ratings from multiple encounters and reducing unwanted variance through adjustments for, by way of example, rater stringency across encounters [24]. Assessment development using ECD, therefore, can help in two ways. First, ECD can provide a structured way for understanding an assessment process at a descriptive level. Second, ECD pushes developers to consider operational concerns, and in particular, the potential need to combine multiple observations to address issues of construct underrepresentation.

### Implementation Research

Central to any assessment system that draws on data directly from practice are the ways in which collecting data and getting scores to the right people to support educational decision-making interacts with preexisting routines [25]. Implementation research, and implementation science more generally, offers a variety of frameworks for clarifying factors affecting the use of evidence-based innovations. In educational contexts, there are multiple factors that need to be considered, such as the readiness of individual faculty members, the availability of technical infrastructure, and the preexisting routines of work environments [18]. Oftentimes, the implementation of assessment systems is taken-for-granted; an explicit focus on implementation can help ensure higher quality data is collected and interpretable score reports are delivered.

Implementation research is a large, complex, and growing body of research, and many frameworks exist [10,26,27]. Table 1 provides a handful of questions that could serve as a preliminary checklist for helping to identify potential issues around the implementation of a technology-based assessment system anchored in collecting data from clinical settings. Non-specific answers to *each* of these questions are potential points of failure in the assessment process and threats to validity in how resulting scores are used. Table 1 is loosely organized by known implementation determinants, such as the design of the innovation, current capabilities of implementers, perceptions and attitudes of implementers, existing policies of the organization, and resources for supporting ongoing implementation.

**Table 1:** Sample Technology and Implementation Questions.


Design: What problem is technology trying to solve? Is the theory of learning well aligned with the assessment process and goals of the medical education program? Capabilities: Is the program ready to adopt a technological-based assessment tool? Is there capacity to take on something new in the HPE system? How well trained are supervisors and trainees in using the technology? Perceptions: How will potential users make sense of the technology—useful or just another “new” thing? How does the technology align with how learners *learn*, how teachers *teach*, and the pre-existing practices of the specialty? Policies: What policies are in place that could prevent individuals from using the technology well (e.g., wi-fi connectivity)? How does the assessment process conflict with ongoing work routines? How does the technology-based assessment fit within the larger program of assessment? Resources: Are there robust social networks within the program to help users problem solve as issues emerge?


### AI Governance

Recent prospective studies have highlighted the potential for artificial intelligence (AI) systems to improve diagnostic accuracy, clinical outcomes, and productivity, and their use is now recommended by national and international clinical guidelines [28,29]. However, the proliferation of AI systems across healthcare systems has also uncovered evaluation and implementation failures that has led to calls for AI governance [30]. While the development of AI tools to support educational decision-making lags behind that of clinical decision-making, AI tools are increasingly used to process and act on data collected from clinical activities for assessment purposes [31].

Therefore, clinical AI governance models can help in calling attention to the ways in which data collected by various technologies that are more proximal to practice in HPE are (1) processed by AI systems and (2) how different systems automatically recommend or enact interventions based on those data [31,32]. Developing AI governance models can help in highlighting a need to more rapidly evaluate both an AI system’s performance and the potential harms of aligned decisions and actions. Assessing consequential validity has long been a practice of traditional assessment development; however, the rapidly evolving algorithmic landscape and the increasing automation of decision-making means that more attention may need to be paid by users of assessment systems that leverage AI [33].

Digital learning environments in HPE are not all that dissimilar from those in other areas of education where a learner interacts with a digital environment, the user generates data through their interactions, and pathways through the environment can vary based on different interaction patterns. Whereas an 8th grade learner may use a digital tutoring system in their development of knowledge, skills, and abilities in algebra, a radiologist or dermatologist may use an AI-driven tool to provide feedback and scaffold learning around their ability to assess medical images [34]. While natural language processing (NLP) has been used for many years in the scoring of written essays in K-16 and professional education, in HPE, uses have expanded to scoring faculty members’ feedback provided to learners as well as the evaluation of trainees’ clinical and discharge notes [35]. For assessment purposes in HPE, models trained on a large number of evaluation ratings are used to predict future performance, and in the case ACGME Milestones, future ratings [36]. Beyond these existing uses, new models are beginning to emerge in areas like medical school admissions [37]. Therefore, as with K-16 education, uses for AI tools are expanding and moving into areas that imply higher and higher stakes for the learner.

As stakes increase and data collection, analysis, and reporting pipelines become more complex, there is an increasing need to evaluate these interconnected systems. Efforts to govern clinical AI combined with developing scholarship in HPE can be used to develop structures for providing oversight of the ways in which data collected from the clinical environment are analyzed using various AI technologies—paying particular attention to the decisions and actions informed by or directly taken by a system [31,32]. The University of Michigan Medical School is developing a governance approach that involves both initial and recurring steps: Initial governance steps entail (1) organizing decision-makers to prioritize educational AI use-cases and (2) setting up technical capabilities to evaluate AI tools. With the proper resources in place, recurring governance activities include (1) piloting, (2) implementing, and (3) evaluating AI tools over time to understand how well the algorithm performs within a local setting and whether an intervention tied to an algorithm is effective. Therefore, AI governance can provide both the motivation and the processes that HPE systems could use to monitor the process of collecting and acting on data generated from clinical practice.

## Conclusion

Many new sources of assessment data are being collected from healthcare settings where learners are directly working with patients. Moreover, new approaches make analyzing these data easier than ever before. In the face of all the various opportunities brought on by new technologies, frameworks from assessment development, implementation research, and AI governance can help in shaping the Next Era of Assessment by providing guidance and successful patterns on which to build new assessment policies and practices.
